# The current applications of cell-free fetal DNA in prenatal diagnosis of single-gene diseases: A review

**DOI:** 10.18502/ijrm.v20i8.11751

**Published:** 2022-09-06

**Authors:** Mohamad Mahdi Mortazavipour, Reza ‎Mahdian, Shirin Shahbazi

**Affiliations:** ^1^Department of Medical Genetics, Faculty of Medical Sciences, Tarbiat Modares University, ‎Tehran, Iran.; ^2^Molecular Medicine Department, Pasteur Institute of Iran, Tehran, Iran.‎

**Keywords:** Cell-free nucleic acids, Prenatal diagnosis, Noninvasive prenatal testing, Single-gene diseases, Non-invasive techniques.

## Abstract

Prenatal diagnosis of hereditary diseases has substantially altered the way medical geneticists are helping families affected by genetic disorders. However, the risk of miscarriage and fear of invasive diagnostic procedures may discourage many couples from seeking prenatal diagnosis. With the discovery of maternal plasma cell-free fetal DNA, prenatal diagnosis has entered a new era of progress. Cell-free DNA is released during normal physiological functions as well as through the cell death programs of apoptosis and necrosis. It can be found in the plasma and other body fluids. Although this method has the advantage of being noninvasive, it is still rather expensive and requires advanced hardware and comprehensive data analysis. Promising implications of noninvasive prenatal diagnosis methods for the diagnosis of common trisomy disorders have paved the way for the development of more complicated assays of single-gene disorders. Relative mutation dosage and relative haplotype dosage are the most widely implemented assays for noninvasive prenatal diagnosis of single-gene disorders. However, each assay has its own advantages and disadvantages. Relative mutation dosage is based on the droplet digital polymerase chain reaction (PCR) technique which includes quantification features of real-time PCR assays. Relative haplotype dosage is based on next-generation sequencing that includes analysis of the maternal and paternal genome followed by sequencing of maternal plasma cell-free DNA. Co-amplification at a lower denaturation temperature PCR is another approach that is based on forming heteroduplexes between alleles to selectively amplify paternal mutations. In this review, we have described the most common noninvasive prenatal diagnosis approaches and compared their applications in genetic disorder diagnosis with different inheritance patterns.

## 1. Introduction

### Cell-free fetal DNA (cfDNA)

Cell-free DNA or cfDNA is released into the plasma following a variety of normal physiological functions such as the cell death programs of apoptosis and necrosis (1). The release of this double-stranded DNA is not limited to the plasma and may occur in other body liquids such as urine or cerebrospinal fluid. Since cfDNA has a fragmented structure, its half-life is very short ( < 2.5 hr); as a result, it has a fast turnover with rapid plasma clearance (2). Increased levels of cfDNA are associated with a range of conditions from infections to inflammation. There is also evidence of elevated plasma cfDNA levels with increasing age. However, cfDNA has been most widely studied in cancer cases and has been successfully used for the evaluation of tumor progression (3). It is also applicable in other pathogenic cell proliferation conditions such as endometriosis (4). Currently, cfDNA molecular profiling techniques are employed by noninvasively identifying somatic mutations; thus precise monitoring of minimal residual disease in different cancers can help to provide appropriate clinical management (5).

Another promising application of cfDNA is in prenatal diagnosis. Lo et al. showed that maternal plasma is an unequal combination of fragmented fetal DNA molecules and a large amount of maternal cfDNA. With the new knowledge about cfDNA, prenatal diagnosis has entered a new era of progress (6). Before the availability of cffDNA, the first-line prenatal diagnosis in medical genetics centers included invasive methods such as chorionic villus sampling, amniocentesis, and cordocentesis. The risk of miscarriage and anxiety from invasive procedures should always be considered as they may discourage couples from undergoing prenatal diagnostic tests (7).

By identifying the characteristics of cffDNA, non-invasive prenatal diagnosis (NIPD) was able to be implemented in clinics (8). NIPD was first applied for the screening of chromosomal aberrations focusing on chromosomes 21, 18, and 13 aneuploidy and identification of sex chromosomes. It is now claimed that NIPD after the 10 ${}^{\text{th}}$ wk of pregnancy has a detection rate of 99.4% and a false-positive rate of 0.16% for trisomy 21. For trisomy 18 the values are 96.6% and 0.05%; however, for trisomy 13 and monosomy X the detection rates are lower (86.4% and 89.5%, respectively) (9). NIPD screening of these major trisomies is based on deep sequencing techniques, although it requires confirmatory invasive testing due to the possibility of placental mosaicism. Microdeletions can also be detected using the chromosomal microarray technique. Both deep sequencing and microarray methods identify the areas of the genome with copy number variation. Detection of specific fetal mRNA expression is another way of screening for aneuploidies. Placenta-specific protein 4, located on chromosome 21, was one of the genes to be applied in trisomy 21 analysis. For detection of Edward's syndrome, the expression of the serpin peptidase inhibitor, clade B, membrane 2 gene, located on chromosome 18, has been employed (10). NIPD diagnostic applications were later expanded to Rh Blood Group D Antigen (RhD) evaluation and fetal sex determination (11). Ultimately, cffDNA has been proposed for NIPD of single-gene diseases. Early uses were focused on the detection of fetal de novo variants or paternally inherited gene mutations. Researchers are currently validating the diagnostic application of cffDNA for a variety of genetic diseases.

Here, we have reviewed the most relevant NIPD approaches that are available for common genetic disorders with an emphasis on single-gene diseases, including their advantages and levels of accuracy.

### NIPD application in single-gene disorders

From the 7 ${}^{\text{th}}$ -9 ${}^{\text{th}}$ wk of gestation, the cffDNA makes up 10% of the circulating DNA in the mother's plasma which is sufficient for a variety of fetal molecular diagnoses. It should be noted that this quantity of DNA is still very low, which makes the implementation of NIPD for single-gene disorders very challenging. Also, since the X chromosome of a male fetus is similar to one of the mother's Xs and has the same haplotype, it can be difficult to discriminate between maternal and fetal genomic variants in X-linked diseases. Additionally, in autosomal recessive disorders, prenatal diagnosis becomes more complicated when the parents have the same mutation with the same haplotype (12). Therefore, various innovative methods have been introduced to perform genetic analysis of cffDNA samples. These methods are mainly based on allele discrimination assays or relative allele dosage analyses (Table I).

**Table 1:** NIPD studies based on detection/exclusion of causative mutations for autosomal disorders


**Author, year (Ref)**	**Pathology**	**Inheritance**	**Methods**	**Sensitivity (%)**	**Specificity (%)**
**Satio ** ***et al.*** **, 2000 (13)**		RFLP	NA	
**Li ** ***et al.*** **, 2007 (14)**		MALDI TOF	NA	
**Lim ** ***et al.*** **, 2011 (15)**		QF-PCR	NI	
**Chitty ** ***et al.*** **, 2015 (16)**		NGS	96.2	100
**Orhant ** ***et al.*** **, 2016 (17)**		dPCR + minisequencing	100	100
**Vivanty ** ***et al.*** **, 2019 (18)**	Achondroplasia	AD & de novo	HRM + minisequencing	100	100
**Van den Oever ** ***et al.*** ** 2015 (12)**				HR-MCA	NI	
**Gonzalez-Gonzalez ** ***et al.*** ** 2003** **(19)**			Huntington's disease	AD (dynamic mutation)	QF-PCR	NI	
**Gonzalez-Gonzalez ** ***et al.*** ** 2008** **(20)**				STRs analysis	NI	
**Amiciacci ** ***et al.*** **, 2000 (21) **			Myotonic dystrophy	AD (dynamic mutation)	PCR & dot blot	NA	
			Hemophilia	XR	Droplet digital PCR	99.8	99.8
	Ornithine transcarbamylase deficiency	XR	Droplet digital PCR	99.8	99.8
	DFNB1	AR	Droplet digital PCR	99.8	99.8
	Acetylcholine receptor deficiency	AR (compound heterozygote)	Droplet digital PCR	99.8	99.8
**Camunas-Soler ** ***et al.*** **, 2018 (22)**	Mevalonate kinase deficiency	AR	Droplet digital PCR	99.8	99.8
	**Camunas-Soler ** ***et al.*** **, 2018 (22)**	AR (compound heterozygote)	Droplet digital PCR	99.8	99.8
	**Hill ** ***et al.*** **, 2015 (23)**		NGS	100	100
	**Nasis ** ***et al.*** **, 2004 (24) **		Allele-specific PCR	100	100
	**Bustamante-Aragones ** ***et al.*** **,** **2008 (25)**		Minisequencing	NI	
	**Guissart ** ***et al.*** **, 2015 (26)**	Cystic fibrosis	AR	MEMO qPCR	NI	
	NIPD: Non-invasive prenatal diagnosis, AD: Autosomal dominant, XR: X-linked recessive, AR: Autosomal recessive, NA: Not applicable, NI: Not indicated, RFLP: Restriction fragment lentgh polymorphism, MALDI TOF: Matrix assisted laser desorption ionization time of flight, QF-PCR: Quantitative fluorescent polymerase chain reaction, PCR: Polymerase chain reaction, NGS: Next generation sequencing, dPCR: Digital polymerase chain reaction, HRM: High resolution melting, HR-MCA: High-resolution melting curve analysis, DFNB1: Nonsyndromic hearing loss and deafness, STRs: Short tandem repeats, MEMO: Mutant enrichment with 3 ' -modified oligonucleotides, qPCR: Quantitative polymerase chain reaction					

#### New diagnostic approaches: Relative mutation dosage (RMD) and relative haplotype dosage (RHD)

RMD analysis is mainly based on the droplet digital polymerase chain reaction (PCR), a technique that is comparable to conventional PCR while including quantification features of real-time PCR. The main technical concept of this method is diluting DNA samples as low as one copy of the genome per micro-unit droplet (13). The micro-units could contain one or no copy of the template DNA. Micro-unit droplets are prepared using water-in-oil emulsion or micro-channel chips (27). This level of dilution accounts for the increased sensitivity and specificity of the PCR reaction by reducing competition effects of target DNA molecules (11). Each digital PCR micro-unit contains 6 nL of reagent mixture which is the minimum volume required for successful PCR amplification. PCR reactions are performed in a real-time PCR apparatus where the amplification of each variant on the target DNA can be detected by a particular fluorophore (28). Recently, researchers have started implementing this assay for NIPD of single-gene disorders. The interpretation of the results depends on the inheritance pattern of the disorder, which is explained in more detail below.

RHD is a next generation sequencing-based NIPD technique, involving maternal and paternal whole-genome sequencing followed by the analysis of cffDNA. The characteristics of the affected fetus are determined by estimating the proportion of mutated to normal alleles. The advantage of RHD over RMD is that with RHD it is possible to examine areas where direct PCR is not possible due to pseudogenes, rearrangements, and complex mutations. However, the problem with RHD is that it requires samples of the proband and his/her siblings. Other limitations of RHD are the high costs of whole exome sequencing (WES) and the complexities of the associated bioinformatics data analysis (29).

To overcome these challenges, targeted locus amplification (TLA) of genomic regions around mutations that harbor informative single nucleotide polymorphisms (SNPs) has been considered. These linked SNPs that are inheritable from one parent have known SNP haplotypes (16). The main purpose of this method is to determine the paternal or maternal origin of the allele. Therefore, it is a successful strategy to find maternal mutations in the fetus which has always been challenging (30). Following targeted sequencing of the desired regions of the maternal and paternal genome, parental haplotypes are mapped to relevant haplotype data obtained from cffDNA (31). TLA has the ability to analyze multiple genomic regions, which is an advantage for the simultaneous detection of multiple single-gene disorders. It will not only reduce costs but also help in cases where the first pregnancy is examined and there is no previous patient sample available. TLA can also be used in trinucleotide repeat expansions that are challenging to be investigated by other methods (29).

### Sex determination approaches and X-linked recessive inheritance

**Figure 1:**
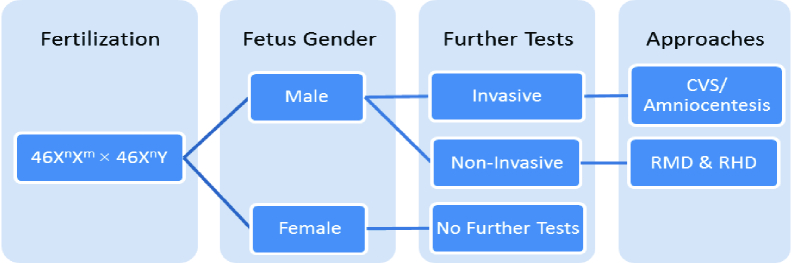
Flowchart of prenatal sex determination for a pregnancy at risk of X-linked disorders. Mutations in the genes located on the X chromosome can lead to an X-linked disorder that is predominantly exhibited in male fetuses, while females are either carriers or unaffected. Therefore, a female fetus needs no more evaluation while the mutation status in a male fetus should be determined by either invasive or non-invasive methods. CVS: Chorionic villus sampling, RMD: Relative mutation dosage, RHD: Relative haplotype dosage.

Mutations in the genes located on the X chromosome can lead to an X-linked disorder that is predominantly exhibited in male fetuses, while females are either carriers or unaffected. In this regard, sex determination using cffDNA can prevent unnecessary invasive tests such as chorionic villus sampling in female fetuses (Figure 1). While NIPD sex determination has reliable results between the 7 ${}^{\text{th}}$ -12 ${}^{\text{th}}$ wk of gestation, conventional ultrasound often is unable to detect the fetus' sex earlier than the 13 ${}^{\text{th}}$ wk of gestation (18). The presence of Y-specific chromosomal sequences in maternal plasma logically indicates male-bearing pregnancies (32). Based on this, studies have been conducted to determine the reliable Y-chromosomal target sequences for non-invasive sex determination (33, 34, 35). The real-time PCR assay has been widely applied to detect Y-chromosomal sequences including *SRY*, *DYS14, DAZ, AMYLY*, and *PAP* genes. The results of a study have indicated a sensitivity of up to 100% at the 8 ${}^{\text{th}}$ wk of gestation (36).

However, complementary molecular tests should be performed to determine whether a male fetus has inherited the maternal causative mutation. These tests should be able to effectively differentiate the fetal mutation in the presence of a large amount of maternal DNA harboring the same allele. In this case, highly sensitive and specific techniques such as RMD and RHD have been implemented for clinically validated assays. In a previous study, RMD analysis was able to detect maternal mutations causing X-linked abnormalities in male fetuses (22).

**Figure 2:**
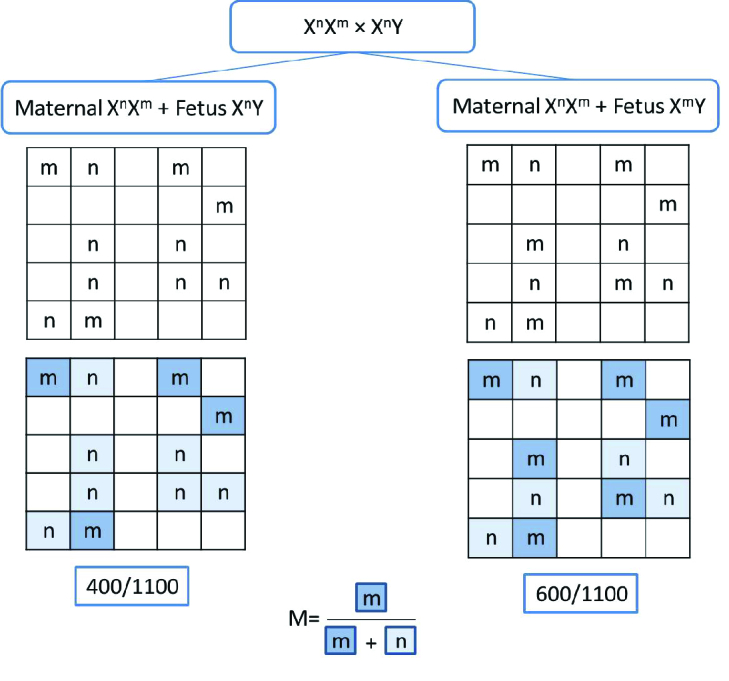
Flow chart of detecting fetal mutations in maternal plasma using the RMD technique in X-linked disorders. Maternal plasma may contain maternal X ${}^{\text{n}}$ X ${}^{\text{m}}$ mixed with either X ${}^{\text{n}}$ Y (normal fetus) or X ${}^{\text{m}}$ Y (affected fetus) genotypes. If the fetus is unaffected, the m/m+n ratio is less than 0.5 while the affected fetus displays a ratio higher than 0.5.

The inheritance of X-linked causative mutations in an at-risk pregnancy (X ${}^{\text{n}}$ X ${}^{\text{m}}$ × X ${}^{\text{n}}$ Y) could result in either an affected fetus (X ${}^{\text{m}}$ Y) or a normal fetus (X ${}^{\text{n}}$ Y). Thus, the maternal plasma may consist of maternal X ${}^{\text{n}}$ X ${}^{\text{m}}$ mixed with either X ${}^{\text{n}}$ Y (normal fetus) or X ${}^{\text{m}}$ Y (affected fetus) genotypes. To determine the fetus genotype, maternal plasma is diluted and transferred into micro-units as a water-in-oil emulsion or micro-channel chips. Statistically, each micro-unit may contain either one or no copy of the template DNA. Each micro-unit contains all the essential reagents for a PCR reaction which is performed in a real-time PCR instrument. 2 distinct TaqMan probes are designed for normal and mutant alleles which are labeled with different dyes such as VIC and FAM. Amplification signals are emitted if a micro-unit contains template DNA. The emitted signals are collected and an amplitude diagram of each dye is drawn for every micro-unit. The mutant allele ratio (M) is calculated by using the formula M = m / m + n (Figure 2).

It is assumed that a heterozygote non-pregnant woman has an M proportion of 0.5 (16). M values > 0.5 indicate an affected fetus whereas non-affected samples display M values < 0.5. The M and fetal DNA fraction data are analyzed by the sequential probability ratio test. The interpretation of RMD is rather convenient as it needs specific primers and probes for every particular mutation (30).

**Figure 3:**
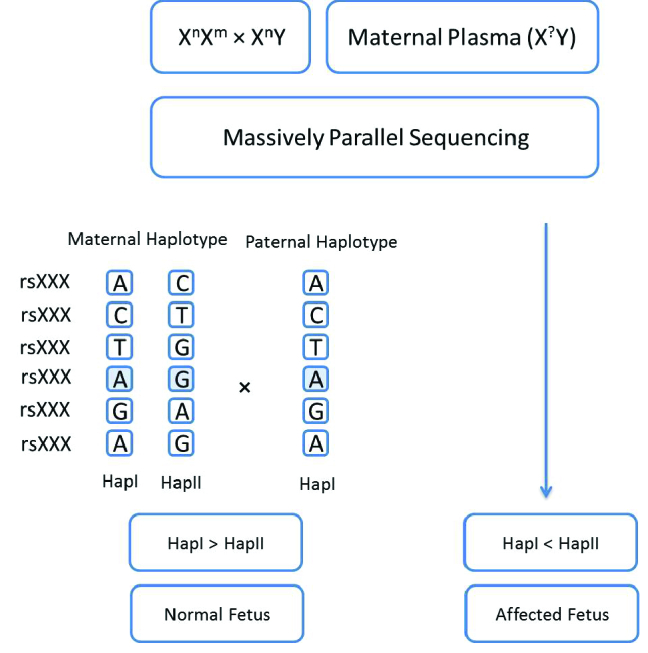
Flow chart of detecting fetal mutations in maternal plasma using the RHD technique in X-linked disorders. Implementation of the RHD analysis for an at-risk pregnancy (X ${}^{\text{n}}$ X ${}^{m}\times$ X ${}^{\text{n}}$ Y) can be performed using massively parallel sequencing to determine the maternal and paternal haplotypes. The desired method can be either whole-genome sequencing or TLA.

RHD can be considered as an alternative approach to detect maternal mutations in male fetuses. As shown in figure 3, haplotype-I is considered as normal haplotype and haplotype-II as the mutated one. The genomic data obtained from maternal plasma cfDNA samples are collected and analyzed in parallel to corresponding genomic data of the parents. The relative quantity of haplotype-I and haplotype-II are determined and if the haplotype-I amount is greater than that of haplotype-II, the fetus might be considered normal, whereas a higher relative quantity of haplotype-II indicates that the fetus might be affected.

### Autosomal dominant disorders

**Figure 4:**
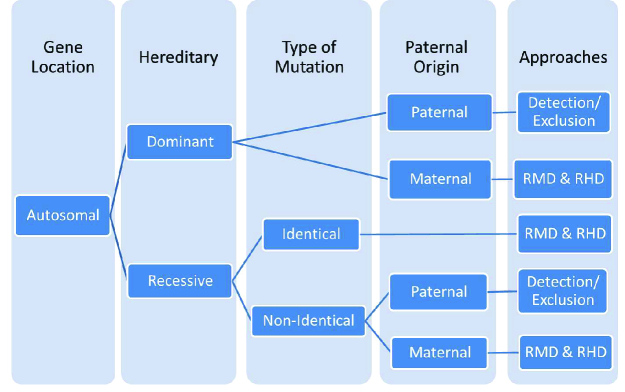
NIPD approaches for genotyping of fetuses at risk of autosomal inherited disorders. RMD: Relative mutation dosage, RHD: Relative haplotype dosage.

In autosomal dominant inheritance, detecting mutations of fetal origin in maternal plasma indicates an affected fetus. As shown in figure 4, there are 2 different approaches for the implementation of NIPD in maternally or paternally inherited autosomal dominant disorders. The detection/exclusion of paternal mutations in maternal plasma determines the status of the fetus in at-risk pregnancies (37). So far, various procedures have been developed for the detection/exclusion of paternal mutations in maternal plasma (Table I). In contrast, the differentiation of fetal alleles is very challenging for maternally inherited mutations. To overcome this limitation, RMD and RHD methods have been successfully applied. In both methods, an equal quantity of mutated and wild-type alleles represents an affected fetus.

However, these methods are not applicable in some autosomal dominant disorders such as Huntington's disease and myotonic dystrophy. These diseases are mainly caused by dynamic mutations classified as trinucleotide repeat expansions. Given that the fragmented fetal DNA in plasma measures about 150 bp, the detection of paternal allele expansions may need alternative methods. For example, the trinucleotide expansions of myotonic dystrophy and Friedreich's ataxia consist of more than 50 and 200 repeats, respectively (5). To this end, quantitative fluorescent PCR (19), TLA (31), and PCR plus fragment analysis (12) have been used for the detection of paternal expansions in maternal plasma. TLA is appropriate for pregnancies in which either paternal or maternal repeats are identical or paternal repeats are much longer than their maternal counterparts (20). Nevertheless, to improve the analysis accuracy, these approaches may be designed exclusively for particular inheritance patterns.

### Autosomal recessive disorders

As displayed in figure 4, NIPD for autosomal recessive disorders has been divided into 2 categories according to the type of the parents' mutations; when the parents have different mutations the fetus is at risk of being compound heterozygote and detection/exclusion strategies are applicable for maternal plasma in these cases (8). If paternal mutations are detectable in the maternal plasma, invasive procedures should be performed to confirm whether the fetus inherited a maternal mutation or not. In contrast, the absence of paternal mutations in maternal plasma indicates that the fetus is unlikely to be affected, and therefore further unnecessary invasive tests can be avoided.

Table I shows various detection/exclusion strategies that have been used to detect autosomal recessive disorders. Recently, co-amplification at a lower denaturation temperature PCR (COLD-PCR) has been described as an innovative detection/exclusion procedure. In brief, the assay exploits melting temperature differences between paternal and maternal alleles based on forming hetero-duplex PCR fragments. In COLD-PCR, critical denaturation temperatures lower than the melting temperature of homo-duplexes are set to selectively amplify paternal alleles (38).

Galbiati et al. reported full concordance between COLD-PCR and microarray techniques as 2 independent, highly sensitive detection/exclusion approaches. However, despite advantages such as cost-effectiveness and rapidity, both COLD-PCR and microarray are limited to NIPD for parents with different mutations (39). RMD or RHD approaches have been successfully implemented when parents have the same mutation (5).

**Figure 5:**
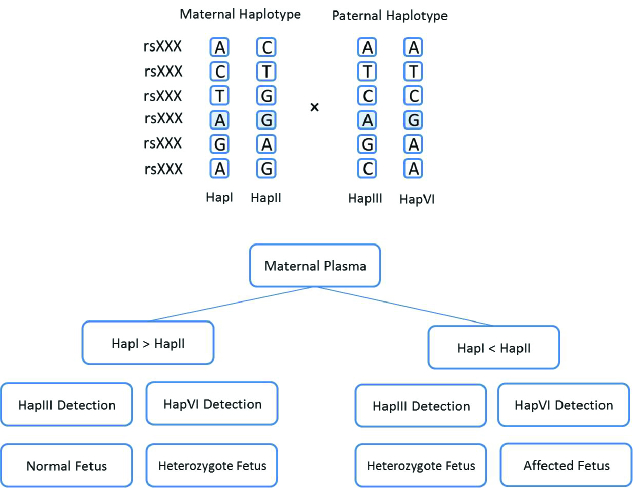
RHD implementation for genotyping of fetuses at risk of autosomal recessive disorders. In non-pregnant female mutation carriers, maternal plasma cell-free DNA consists of an equal proportion of haplotype-I and haplotype-II, which are considered normal and mutant haplotypes, respectively. In the 10 ${}^{\text{th}}$ wk of pregnancy, fetal cell-free DNA is mixed with the maternal haplotypes and changes haplotype-I and haplotype-II proportions. The interpretation of the results depends on the detection of paternal haplotypes haplotype-III or haplotype-IV.

In RMD, a balanced proportion between mutant and normal alleles in the maternal plasma indicates a heterozygote fetus whereas an imbalanced allele proportion may represent either a normal or affected homozygote fetus (37). An example of RHD or TLA interpretation for NIPD of autosomal recessive cases is shown in figure 5. An archetype of the autosomal recessive diseases commonly proposed for NIPD is beta-thalassemia, especially in the areas where the disease is common (40).

Given the cost and complexity of the management of beta-thalassemia, prenatal diagnosis is recommended to at-risk families (41, 42). Some countries such as Italy, Greece, Cyprus, and Iran have developed screening programs for carrier detection and prenatal diagnosis (40). More than 200 distinct mutations in the *HBB* gene involving single base mutations, small and large deletion, and aberrant splicing mutations can lead to the clinical features of the disorder (41).

Considering the autosomal recessive inheritance pattern of this disease, NIPD for beta-thalassemia can be implemented according to the parent's mutation types (43). RMD and RHD approaches have been recommended for parents that share similar mutations (22, 44, 45).

Furthermore, detection/exclusion strategies can identify a paternal mutation in maternal plasma in compound heterozygote cases (46, 47). Investigation of 75 beta-thalassemia carriers for 2 common mutations (Cd39 and IVSI.110) using full COLD-PCR showed promising results in a study conducted in Italy (39). We also successfully developed a COLD-PCR-based method to detect the paternal beta-thalassemia IVS-II-1 (G > A) mutation in maternal cell-free DNA (48). Furthermore, another study showed that fast temperature-gradient COLD-PCR was able to identify 2 paternal SNPs in maternal plasma samples (38). The studies which have reported the results of NIPD of beta-thalassemia as an archetype of single-gene disorders are summarized in table II.

**Table 2:** NIPD approaches implemented for determining fetal genotype in beta-thalassemia


**Author, year (Ref)**	**Mutations**	**Methods**	**Sens.**	**Spec.**
**Li ** ***et al.*** **, 2005 (49)**	*IVSI*-1, *IVSI*-6, *IVSI*-110, and codon 39 (compound heterozygote)	Peptide-nucleic-acid clamp and allele-specific real-time PCR	100	93.8
**Galbiati ** ***et al.*** **, 2016 (39)**	*Cd39 and IVSI.110*	COLD-PCR and microarray	100	100
**Mortazavipour ** ***et al.*** **, 2020 (48)**	*IVSII-1*	COLD-PCR	NA	
**Phylipsen ** ***et al.*** **, 2012 (50) **	12 different fragments along with the β-globin gene cluster	PAP and MCA	NI	
**Papasavva ** ***et al.*** **, 2008 (51) **	11 SNPs	APEX method	NI	
**Yi ** ***et al.*** **, 2010 (52)**	*IVSII-654 (C → T)*	PCR/LDR/capillary electrophoresis	NI	
**Yi ** ***et al.*** **, 2010 (53) **	*CD17 (A → T)*	PCR/LDR/capillary electrophoresis	1:5000	NI
**Chiu ** ***et al.*** **, 2002 (54)**	*41/42 (–CTTT)*	Real-time PCR	100	100
**Lun ** ***et al.*** **, 2008 (44) **	*CD41/42 (–CTTT* ***)***	RMD & NASS	NI	
**Li ** ***et al.*** **, 2009 (55) **	*Cd39*	Mutation-specific PCR & MALDI TOF	NA	
**Ramezanzadeh ** ***et al.*** **, 2016 (56)**	*CD44, IVSI-1, FR8*-9*, and IVSI-5*	Allele-specific real-time PCR	100	100
**Wang ** ***et al.*** **, 2017 (45)**	*CD41-42 (-TTCT)*	RHD	100	100
**Camunas-Soler ** ***et al.*** **, 2018 (22)**	Droplet digital PCR (RMD)	99.8	99.8
**Chan ** ***et al.*** **, 2010 (57)**	4 SNPs in connection with* HBB *gene	AS-APEX	100	100
**Byrou ** ***et al.*** **, 2018 (38)**	2 SNP*s (rs7480526 and rs968857)*	Fast TG COLD- PCR	NA	
**Yenilmez ** ***et al.*** **, 2013 (46)**	Used 4 primers: P1:*Cap +22 (G > A) * and*-30 (C > T)* P2: *IVSI-1 (G > A), IVSI-5 (G > A), IVSI-6 (T > C), IVSI-110, (G > A), Cd8 ( - AA), Cd9/10 (+T), Cd15 (G > A), *and* Cd39 (C > T) * P3: *IVSII-1 (G > A) * P4:* IVSII-745 (C > G) *and* IVSII-848 (C > A)*	HRM	100	100
**Saba ** ***et al.*** **, 2017 (47)**	*c.118C4T*	Semiconductor sequencing	100	100
**Lam ** ***et al.*** **, 2012 (29)**	*Cd41/42, 28(A > G), *and* CD17*	MPS	100	100
NIPD: Non-invasive prenatal diagnosis, Spec: Specificity (%), Sens: Sensitivity (%), NA: Not applicable, NI: Not indicated, PAP: Pyrophosphorolysis-activated polymerization, MCA: Melting curve analysis, SNPs: Single nucleotide polymorphisms, LDR: Ligase detection reaction, NASS: Nucleic acid size selection, MALDI TOF: Matrix assisted laser desorption ionization time of flight, RMD: Relative mutation dosage, RHD: Relative haplotype dosage, AS: Allele specific, APEX: Arrayed primer extension, TG COLD-PCR: Temperature gradient coamplification at lower denaturation temperature PCR, HRM: High resolution melting, MPS: Massively parallel sequencing, PCR: Polymerase chain reaction				

## 2. Conclusion

Recent advances in NIPD for the identification of single-gene diseases have provided promising prospects in prenatal diagnosis. We can expect that in the coming decade, a significant number of single-gene disorders will be detected by NIPD methods. However, ethical issues are among the concerns that should be addressed before the widespread implementation of advanced NIPD techniques. Ultimately, population-specific genome databases are highly desired for their high specificity in the detection of mutations within the context of inter-population variation.

##  Conflict of Interest 

The authors declare that there is no conflict of interest.
